# The Medical Department of the Army and Its Critics

**Published:** 1898-09

**Authors:** 


**Monthly Review of Medicine and Surgery.**

*EDITORS:*

THOMAS LOTHROP, M. D. - - WM. WARREN POTTER, M. D.

All communications, whether of a literary or business nature, should be addressed to the managing editor: 284 FRANKLIN STREET, BUFFALO, N. Y.

VOL. XXXVIII. SEPTEMBER, 1898. NO. 2

### THE MEDICAL DEPARTMENT OF THE ARMY AND ITS CRITICS.

THE yellowest exhibition of yellow journalism since the advent of this ochre-tinted type of literature has been the recent attacks on the conduct of the medical department of the army in its operations in Cuba.

Intimation of what might be expected was given in the first reports sent out after the battle of El Caney, wherein the medical officers were severely condemned for laxity in not providing immediate relief for the wounded soldiers. And, again, before Santiago the correspondents sent out dismal reports of frightful suffering among the wounded for want of surgical assistance.

All of which goes to show the correspondents were simply stumbling along darkly in the rutty road of journalistic ignorance of medical and surgical matters—an ignorance which is as painful as it is inexcusable in these days of enlightenment, when newspapers of progressive spirit employ Generals as experts to write of military operations, retired admirals to write of and describe naval battles, naval constructors to draw diagrams of sunken battle ships and battle plans of the movements of the steel clad war vessels in action. They do all this and more, they employ eminent divines to write of the ills of the soul; they hire chemists to dig into the secrets of city air and sewage, to turn over and analyse free lunch counters and dirty bank bills; and yet, in all this war, ordinary reporters have had " license full and fancy free " to write of and criticise the medical department of the army; to cast slurs upon hygienic management of camps, hospitals and ships, to condemn methods, men and measures, without knowing the rudiments of medicines or understanding the first principles of surgery or sepsis.

The shame deepens on modern journalism when one reads such paragraphs as these, which are taken from daily New York newspaper reports: " The bullet passed through his skull, tearing great holes on each side, from which brains poured. He was operated on and will recover." " The surgeon cut him open and tore his intestines and stomach out. They sewed these up and washed them off and then sewed up the great wound they had made. ——— lay on the table, which was dripping with blood. He opened his eyes and saw the surgeons watching him, their arms red to the elbows with his life's blood, the table dripping with the same fluid, which gathered in great pools underneath; and he smiled and said 'By ——! you can't kill me!' "

Then came the stories of the alleged mismanagement at Santiago and Siboney and on board the transports, which, stranger still to relate, were taken up by Gen. Shafter and referred to in his report to the war department.

Their appetites whetted by the preliminary reports, the newspapers sent men out to meet the incoming transports, the Seneca, the Concho and others, at New York, and there was thrown on the great screen of publicity some harrowing pen pictures of frightful goings on aboard the transports. Men, wounded unto death, so ran the reports, were packed together like sardines; their wounds were seldom dressed and when they were " the washing was done with salt water," owing to an entire lack of fresh water; surgical dressings occupied no place in the ship's equipment. Such was the tenor of the reports and like evil creatures begat in darkness and ignorance they grew until they became ogres of horror and the newspaper-reading public, thrilled by the heroic achievement of the American forces on land and water, paused in their admiration for the boys in blue to cast reproaches on the men who were placed in charge of the lives of the seamen and soldiers; to cast reproaches on the men who were on the firing line, shoulder to shoulder with the infantry, shoulder to shoulder with the Rough Riders; face to face with the enemy, working, slaving, braving death, fearless of danger, unshrinking, unswerving, unshirking. These are the men on whom reproaches were cast. And in far-away Washington there sat a man with a master mind; a man whose life has been devoted to the study of medicine and its allied sciences; a man whose whole aim and ambition has been to care for the soldiers and care for them properly, whose duty it was to care for the sick and wounded, even though the wounds and ills were brought about by what are generally termed "military errors of judgment." On this man, the Surgeon-General, Dr. George M. Sternberg, were heaped reproaches until for the sake of the honor of his corps—not for himself, however, for it will be noticed he does not defend himself—he is forced into a defense, and he makes it in that quiet, dignified, manly way which is so characteristic of the man. Regarding his professional qualifications the JOURNAL needs to say but one word, 'Twere like carrying diamonds to Kimberly to refer to an acknowledged leader as an expert.

From Dr. Sternberg's letter to the *Medical Record*, of New York, which strange to say was one of the foremost in the hypercritical crowd, we quote briefly :

The number of medical officers allowed by law is inadequate. The total number is 192, . . . . This leaves 96 medical officers available for duty with troops in the field. Of these thirty-five have been appointed brigade surgeons of volunteers and are distributed among the various army-corps. . . . .

General Shafter's Army, at Tampa, was thoroughly well supplied with the necessary medicines, dressings, etc., for field service, but owing to insufficient transportation he left behind at Tampa his reserve medical supplies and Ambulance Corps. Every one who has read the papers knows about the difficulties encountered in landing supplies at Siboney. As is usual under such circumstances, the fighting men with their guns and the rations necessary for their subsistence were first landed and hurried to the front. The Relief, loaded to her utmost capacity with medical supplies, arrived at Siboney four days after the fight at El Caney. That she was not able to get there sooner was a great disappointment to me, but was no fault of the Medical Department. I asked for a hospital ship in good time, but there was unavoidable delay in securing a suitable vessel and in preparing her for service.

It has not been the expectation of the Medical Department that every wounded man would immediately receive the attention of a surgeon. No modern army makes provision for so large a number of medical officers as this would require. But attached to our Army there is a Corps of noncombatants known as the Hospital Corps, which is the organised and authorised Red Cross Corps of the Army. At the outbreak of the war we had 800 Hospital Corps men in service. At present there are more than 4,000. These men wear a brassard upon the left arm bearing the Red Cross of the Geneva Convention. We have done our best to instruct them in giving first aid to the wounded, and in a majority of cases a first aid dressing properly applied by one of these men is all that is required. All of the surgeons who have come from the front have testified to the remarkable results attained from the prompt application of aseptic dressings by our Hospital Corps men and by the soldiers themselves or their comrades. The proper application of the dressings contained in the first aid packet, which is carried by every soldier, is, under existing regulations, a matter in which every enlisted man has special instruction. . . . .

The first aid dressing is to be put on at or near the firing line, and the men have usually to be transported to the field hospitals on litters. In this age of aseptic and conservative surgery comparatively little operating is required, and our regular force is ample to attend to this. Moreover, we have men selected for their experience and skill to decide whether operations are necessary and to perform them. . . . .

If there has been any failure to get necessary supplies to Cuba it has been because of the difficulty of securing adequate transportation and of landing supplies through the surf; but we have been constantly importuned to transport Red Cross agents and female nurses to the front. I am informed that many of these so-called nurses had never received any special training to fit them for the duties they were so eager to undertake.

As regards the Seneca, I am informed by Major Torney, in charge of the Hospital Ship Relief, that he transferred forty-two (42) wounded men, most of whom were entirely able to care for themselves, from the Relief to the Seneca, because at that time it was thought our troops would storm Santiago and that a large number would be sent to the hospital ship for treatment. . . . . Two acting assistant surgeons were sent on board with a supply of the most necessary medicines and dressings. . . . . When the medical officers now at the front return home I expect to have full information with reference to the efficiency of the medical service in the vicinity of Santiago, and in the meantime I hope the profession at large will not be so ready to draw inferences unfavorable to the Medical Department of the Army on the basis of reports published in the daily papers as the editor of the *Medical Record* has been.

There is no doubt the medical department was hampered by the failure to move its supplies; but what would these self-same newspapers have had to say if the reverse action had taken place? Suppose the army had been of secondary importance and the medical department and its supplies had been sent to the battle-field first, in order that everything might be ready for the first man who fell. If this had been done the flag of blood and fire would have been floating from the staff where now flies that emblem of succor and mercy, the red cross. There may have been mistakes. It is easy for the non-combatant many, many miles away from the battle-field and ignorant of the topography of the country and the formation or position of the enemy, to tell what he would have done; it is easy for him to pick flaws in the conduct of operations in which half a million men are concerned. Such men are carpers, self-inflated, presumptuous and opinionated. They see no reason why a wounded man should not receive immediate attention; they see no reason why surgeons should have such "soft snaps," forgetting in their inflated conceit that they are living in a peaceful city, where the accidental discharge of a toy revolver would set hearts to thumping with apprehension and fill every window in the neighborhood with a frightened face. And the shame of it all is that these false reports were circulated by newspapermen who were on the ground and who knew, or should have known that war makes its own laws.

The JOURNAL does not defend Dr. Sternberg or the medical corps for the simple reason that they need no defense. The duty of that department has been well done. The army went ahead and made its conquests. The surgeons went with the soldiers and performed their duty and made their conquests. And to those critics—the stay-at-homes—who are ever ready and over-anxious to rush in where angels fear to tread, the JOURNAL calls attention to this fact:

Among the first Americans to meet death in the Cuban campaign was a medical officer who was doing his duty.

